# Malaria vaccine coverage estimation using age-eligible populations and service user denominators in Kenya

**DOI:** 10.1186/s12936-023-04721-0

**Published:** 2023-09-27

**Authors:** Angela K. Moturi, Rose Jalang’o, Anitah Cherono, Samuel K. Muchiri, Robert W. Snow, Emelda A. Okiro

**Affiliations:** 1 Population & Health Impact Surveillance Group, KEMRI-Wellcome Trust Research Programme, Nairobi, Kenya; 2 National Vaccines & Immunization Programme, Ministry of Health, Nairobi, Kenya; 3 Centre for Tropical Medicine and Global Health, Nuffield Department of Medicine, University of Oxford, Oxford, UK

**Keywords:** Malaria, Vaccine coverage, RTS,S/AS01, Malaria vaccine pilot, Kenya

## Abstract

**Background:**

The World Health Organization approved the RTS,S/AS01 malaria vaccine for wider rollout, and Kenya participated in a phased pilot implementation from 2019 to understand its impact under routine conditions. Vaccine delivery requires coverage measures at national and sub-national levels to evaluate progress over time. This study aimed to estimate the coverage of the RTS,S/AS01 vaccine during the first 36 months of the Kenyan pilot implementation.

**Methods:**

Monthly dose-specific immunization data for 23 sub-counties were obtained from routine health information systems at the facility level for 2019–2022. Coverage of each RTS,S/AS01 dose was determined using reported doses as a numerator and service-based (Penta 1 and Measles) or population (projected infant populations from WorldPop) as denominators. Descriptive statistics of vaccine delivery, dropout rates and coverage estimates were computed across the 36-month implementation period.

**Results:**

Over 36 months, 818,648 RTSS/AS01 doses were administered. Facilities managed by the Ministry of Health and faith-based organizations accounted for over 88% of all vaccines delivered. Overall, service-based malaria vaccine coverage was 96%, 87%, 78%, and 39% for doses 1–4 respectively. Using a population-derived denominator for age-eligible children, vaccine coverage was 78%, 68%, 57%, and 24% for doses 1–4, respectively. Of the children that received measles dose 1 vaccines delivered at 9 months (coverage: 95%), 82% received RTSS/AS01 dose 3, only 66% of children who received measles dose 2 at 18 months (coverage: 59%) also received dose 4.

**Conclusion:**

The implementation programme successfully maintained high levels of coverage for the first three doses of RTSS/AS01 among children defined as EPI service users up to 9 months of age but had much lower coverage within the community with up to 1 in 5 children not receiving the vaccine. Consistent with vaccines delivered over the age of 1 year, coverage of the fourth malaria dose was low. Vaccine uptake, service access and dropout rates for malaria vaccines require constant monitoring and intervention to ensure maximum protection is conferred.

**Supplementary Information:**

The online version contains supplementary material available at 10.1186/s12936-023-04721-0.

## Background

The malaria burden young children suffer in sub-Saharan Africa (SSA) remains unacceptably high. Despite increasing coverage of vector control and effective, prompt treatment, reductions in the malaria burden have stagnated. The future is threatened by emerging drug resistance, escalating insecticide resistance, and decreasing international donor assistance [1]. New, cost-effective tools are required to mitigate against a resurgence of malaria to pre-2000 levels and accelerate future disease burden reductions. Decades of research have positioned new approaches to malaria control in Africa, including novel strategies for chemoprevention and vaccination [1–3].

The RTS,S/AS01E (RTS,S/AS01) vaccine targets pre-erythrocytic stages of the malaria parasite. Phase III trial results indicated a 39% protection against clinical malaria and a 29% reduction in the incidence of severe malaria among children aged 5–17 months who receive all 4 doses through the expanded programme of immunization (EPI) services [4]. In 2015, RTS,S/AS01 received a regulatory positive opinion approval from the European Medicines Agency [5]. To better understand safety, feasibility, and mortality impact under routine conditions, the World Health Organization (WHO) sponsored a phased implementation in 2019 in Malawi, Ghana, and Kenya, randomizing high malaria burden administrative areas to receive an early implementation of RTS,S/AS01 [6]. Early analysis has shown that three doses of the RTS,S/AS01 vaccine delivered through routine EPI services are safe and feasible, and disease reductions are consistent with Phase III trials [4, 7]. In October 2021, RTS,S/AS01 became the first malaria vaccine to be approved for wide-scale use by the WHO [8]. The success of the RTS,S/AS01 rollout in SSA, as with other proven efficacious interventions, will be assessed using estimates of vaccine dose coverage. Therefore, vaccine coverage measures must provide robust and reliable evaluations at various scales for national and sub-national decision-making.

The gold standard for estimating vaccine coverage is through nationally representative household surveys. However, these are undertaken infrequently, limited to small sample sizes, and are susceptible to selection, recall and information biases [9]. National EPI programmes frequently utilize routine immunization data from national health information systems to evaluate coverage at both national and subnational levels. The standard approach to computing annual vaccine coverage for various antigens is by calculating the proportion of children who receive a vaccine relative to the total projected population of children under 1 year (denominator) [10–13]. However, the effectiveness of this method is hindered by inaccurate estimates of the target denominator population size and a lack of accounting for migrations and health facility catchments [12, 14].

An alternative method that seeks to overcome the challenges of population-based coverage exploits linked health service data, e.g., other vaccines, as the denominator [12, 15, 16]. This approach is applicable for vaccines requiring multiple doses, for which the first dose is considered nearly universal, thereby capturing a large majority of the population who use immunization services. For example, such an analysis might ask what proportion of children who obtain Pentavalent (DTwP-Hib-HepB) (Penta) dose 1 vaccine, go on to receive Measles–Rubella dose 1?

The RTS,S/AS01 vaccine pilot programme in Kenya has been implemented for more than 36 months, establishing a consistent delivery system integrated into the routine immunization schedule. The Kenyan pilot schedule targets children the age of 6, 7, 9, and 24 months for doses 1–4 respectively. The unique schedule of this vaccine, extending beyond the current Kenyan immunization programme timelines and its implementation in selected subnational regions provides an opportunity to understand the challenges and implications associated with introducing a new vaccine.

The objective of the present study was to estimate the coverage of the RTS,S/AS01 vaccine in Kenya from 2019 to 2022 using routine data employing both population and service-based denominators.

## Methods

### Study setting

The pilot RTS,S/AS01 implementation programme in Kenya was undertaken in eight counties [17] in the Western region: Bungoma, Busia, Homa Bay, Kakamega, Kisumu, Migori, Siaya, and Vihiga. Despite intensive vector control intervention since 2010 [18], the eight counties continue to represent the highest malaria transmission counties in Kenya, with many locations showing *Plasmodium falciparum* prevalence in the community of over 30% [19, 20]. The malaria hospital burden in this area remains high, and severe malaria is concentrated in children under 5 years of age [21–23].

### RTS,S/AS01 vaccine implementation

In Kenya, the National Vaccines and Immunization Programme (NVIP) has led a phased implementation of the RTS,S/AS01 malaria vaccine within select sub-counties across the eight counties. The implementation areas were selected using a constrained randomization process that factored in considerations such as a *P. falciparum* prevalence of over 20%, consistently high coverage for other childhood vaccines, and sufficient populations of surviving age-appropriate children (< 1 year) to receive the vaccine. Of the 62 sub-counties in the initial selection, 26 were defined as intervention areas, 23 served as controls while 13 sub-counties did not meet the selection criteria. This study excludes 3 intervention sub-counties that were implementing the vaccine but were already part of a Phase IV trial [7, 24].

The pilot began in September 2019 and, by August 2022, had been implemented for 36 months, the period considered for analysis in this paper. It is important to note that the Kenyan implementation reviewed the age criteria for the first dose of the vaccine to allow children up to the age of 1 year to receive the vaccine. This change was effected in the early months of the rollout but the minimum age of 6 months was retained. Due to the lack of records of the number of children above 6 months who took part in the first year, this study maintains the initial age criteria in subsequent analysis [37].

Vaccinations in the EPI schedule are delivered at no cost to the user at various health facilities in the region. Health facilities are owned and managed by several providers, including the Ministry of Health (MoH), non-governmental organizations (NGO), faith-based organizations (FBO), private-for-profit entities and ‘other’ management such as those for schools, parastatals, military and prison entities that provide services to select populations. All facilities are assigned a Kenya Essential Packages for Health (KEPH)-level designation corresponding to services provided within the facility. KEPH levels reflect increasing service complexity: primary care facilities are classified as Level 2–3 and include clinics, dispensaries and health centres, while secondary care facilities are classified as Level 4–5 and comprise primary and secondary referral hospitals. Tertiary facilities are classified as Level 6 and comprise national referral facilities [25].

### Data

#### Routine EPI data

Careful consideration was given to selecting appropriate service-use denominators for the analysis of vaccine coverage. The selection of vaccine antigens was based on their delivery schedule in relation to the RTS,S/AS01 vaccine schedule in Kenya, which is administered in four doses to children aged 6, 7, 9, and 24 months. The selection process relied on the national routine immunization schedule (Additional file 1: Table S1 as specified by the Ministry of Health [26]. The inclusion criteria comprised vaccines administered before 14 weeks, to determine the baseline of children already receiving immunizations and vaccines given at the end of the routine schedule (9 months and over) which are approximate to RTS,S/AS01 dose 3 and 4 scheduled administration. The vaccine antigens selected include: Bacille Calmette-Guerin (BCG) and Oral Polio Vaccine (OPV1) (given at birth), Pentavalent (DTwP-Hib-HepB) (Penta) 1–3 given at 6, 10 and 14 weeks and Measles-Rubella (MR) 1–2 given at 9 and 18 months.

Immunization data were sourced from District Health Information Software version 2 (DHIS2), the Kenya Health Information System. DHIS2 is an open-source, web-based platform for data collection, analysis, and reporting of health programmes implemented nationally in Kenya since 2011 [27]. Data for the selected vaccines were extracted from DHIS2. The data were downloaded on October 11, 2022, and comprised of facility-level monthly reports of total vaccine antigens administered, disaggregated by antigen and dose for all eight counties in Western Kenya over 41 months, from April 2019 to August 2022. Data for the 5 months preceding the start of RTS,S/AS01 implementation (April to August 2019) were included to capture information on other vaccines administered to children who would have been eligible to receive RTS,S/AS01 dose 1 in September 2019.

Analysis of vaccine antigen coverage was thereafter restricted to health facilities that provided immunization services, defined as one that makes any report, across the period of interest, of administering BCG, OPV1, Penta 1–3, MR1, MR2 and RTS,S/AS01 1–4. Further exclusions were made to remove the few facilities that reported less than five doses of the RTS,S/AS01 dose 1 vaccine across the 36-month observation period. These were assumed to be erroneous entries (Additional file 1: Figure S1).

Each facility report was matched to a recently updated and geocoded national health facility database to obtain comprehensive data on immunizing facilities in the eight counties [28]. This list was developed by sourcing and cross-referencing health facility data from the DHIS2 and the Kenya Master Health Facility List (KMHFL). The facilities in this listing included all operational public and private sector providers that offered curative and preventative health services. This database assembled additional health facility details unavailable within the DHIS2 platform, including, ownership, Kenya Essential Package for Health (KEPH) level and geographic coordinates, confirmed using Google Earth.

#### Computing dropout rate

Multi-dose vaccines routinely experience attrition across the vaccination schedule, frequently termed as dropouts. Dropout is when children receive one or several doses of vaccinations but fail to complete the entire vaccine series. To understand the variable performance across doses, this study calculates the dropout rate across the RTS,S/AS01 vaccine schedule relative to the first dose. This was done by computing the difference between two doses (e.g., doses 1 and 2) and dividing the difference by the number of children who received the first dose (dose 1), expressed as a percentage.

#### Computing vaccination coverage

Vaccine coverage was computed using two approaches: (a) a service-based denominator to obtain coverage metrics among children already engaged in EPI delivery; and (b) a population-based denominator to assess coverage within the overall under-1 target population.

##### Vaccine coverage using a service-based denominator

This approach defines the denominator as the count of all children who have received prior vaccines and are expected to return for follow-up immunizations. The earliest vaccines administered to children, such as BCG or Penta 1, are ideal for obtaining a broad benchmark of children engaged in the EPI programme. However, BCG administration is skewed toward hospitals where delivery services are available and serving populations across several sub-counties. Comparably, Penta 1 is often sought at health facilities closer to households, providing a better estimate of catchment populations within sub-counties. Given the high Penta 1 coverage (99%) within the study area, [29] and its use as a service use denominator in previous vaccination coverage studies [12, 15, 30] this study selected Penta 1 as the service use denominator.Directed by the initial RTS,S/AS01 guidelines on age eligibility for each dose and assuming the receipt of a timely dose of Penta 1 (Additional file 1: Table S1), the number of children who would be eligible for inclusion in the denominator was estimated per month, for each dose of malaria vaccine. The timelines of Penta 1 administration were used to determine the count of eligible children (Additional file 1: Figure S2). For example, to be eligible to receive dose 1 in September 2019, at 6 months of age, a child would have received Penta 1 in April 2019. The number of children who received Penta 1 in April 2019 would thus be the denominator for malaria vaccine dose 1 in September 2019. Using this framework, Penta 1 delivered at various periods corresponding to a child being age-eligible for each dose was used to obtain the denominator. This minimizes the underestimations of coverage due to the inclusion of children, within the denominator, that are yet to qualify to receive a given dose based on age.

Similar to other multi-dose vaccines, RTS,S/AS01 doses 3 and 4 are subject to dropout. To evaluate these later vaccine doses appropriately, additional coverage analysis for doses 3 and 4 was undertaken using MR doses 1 and 2 as denominators to account for expected dropout. This analysis assesses the performance of concurrent vaccine delivery (RTS,S/AS01 dose 3 against MR1) and the uptake of the RTS,S/AS01 dose 4 among children already receiving vaccinations over the age of 1 year such as MR2 for which coverage is lower at approximately 67% nationally [29]. To determine the coverage of RTS,S/AS01 dose 3 using MR dose 1 as a denominator, counts of MR1 doses reported during the same period as the numerator were used given both vaccines are administered at 9 months. To evaluate the coverage of RTS,S/AS01 dose 4 using MR2 as the denominator, the framework described earlier was employed to determine the denominator count. In this case, eligible children in each month were defined as those who had received the MR2 vaccine 6 months prior (Additional file 1: Figure S2).

##### Vaccine coverage using a population-based denominator

To estimate malaria-vaccine dose coverage of all young infants within the 23 sub-counties, the annual total population of children under the age of one for the period spanning 2019–2022 was derived from the publicly available platform, WorldPop [31]. WorldPop employs a combination of official census data, satellite imagery—which includes land cover and night-time lights—and dasymetric modelling techniques to produce population datasets that are disaggregated by broad age groups including infants at a spatial scale of up to 100 m. National population growth data is utilized to forecast population counts for intercensal years [31, 32] and has been utilized in similar vaccination coverage studies [33, 34].

Population counts were extracted for each of the 23 implementation sub-counties using ArcGIS Version 10.5 [35] (Additional file 1: Figure S3). To estimate the population within an RTS,S/AS01 implementation year which spans two calendar years (from September to August), the average population of the two consecutive years was computed and used as the denominator for each implementation year. As finer age classification cannot be derived from annual population estimates (i.e. only children aged ≥ 6 months), it was assumed that the under-1-year population remains a suitable denominator for the first three doses. In the case of dose 4, the denominator used was the preceding year’s total under-1 population that would be turning 2 years i.e., dose 4 denominator for the second year of implementation was the total under-1 population in the first year of implementation.

Coverage for both approaches was assessed at two scales: across the entire implementation area (23 sub-counties) and by sub-county. The numerator was the total monthly counts of administered RTS,S/AS01 vaccines, by dose. Analysis was carried out across the entire 36-month period of RTS,S/AS01 delivery and for each implementation year using Microsoft Excel and Stata SE, Release 17 [36]. This study excluded early delivery of dose 4 (before March 2021) due to revisions made to the age eligibility of dose 1 [37].

## Results

### Vaccinating health facilities

**Fig. 1:**
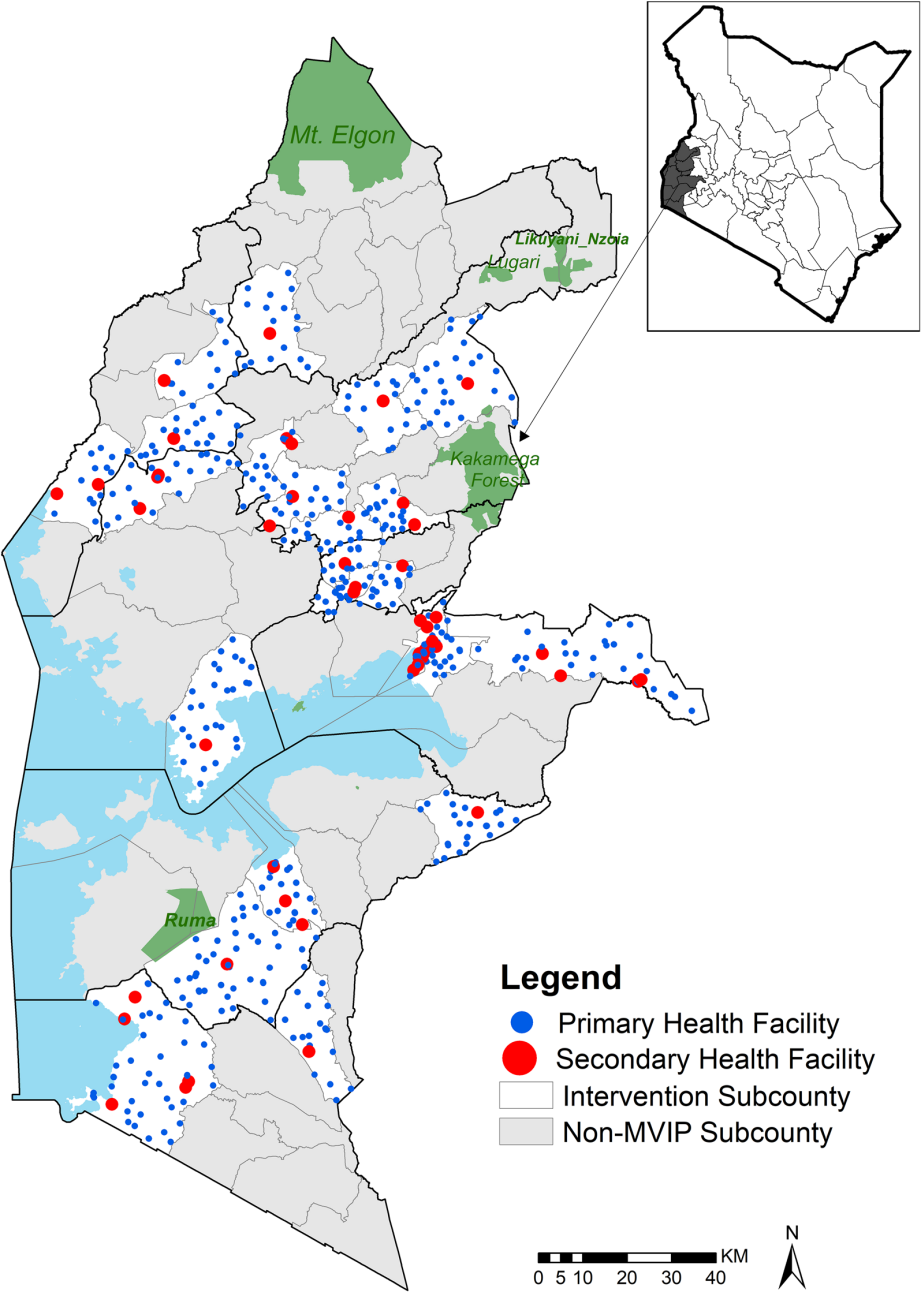
Geographic distribution of vaccinating health facilities across RTS,S/AS01 vaccine implementation sub-counties in Western Kenya (N = 537). Primary care facilities are classified as Level 2–3 and include clinics, dispensaries and health centres while secondary care facilities are classified as Level 4–5 and comprise primary and secondary referral hospitals [25]

Eight hundred and forty-two health facilities were identified from DHIS2 across all 23 malaria vaccine implementation sub-counties. Forty-two (5%) facilities that provided specialist services, 69 (8%) opened/newly registered after September 2019 and 168 facilities that did not make a single report of vaccinations over 36 months were excluded. Twenty-six facilities that reported administering less than five RTS,S/AS01 dose 1 over the 3 years were also exempted (Additional file 1: Figure S1). 537 vaccination facilities were considered in the analysis (Fig. 1), and comprised facilities managed by the Ministry of Health (MoH) (72%), the private-for-profit sector (15%) faith-based (FBO), non-governmental organizations (NGO) and ‘other’ owners (schools, army, parastatals) (13%) (Additional file 1: Table S2). 90% of facilities in these sub-counties are primary facilities (Fig. 1).

### RTS,S/AS01 vaccination delivery

Over the 3 years, the estimated number of eligible children for the service denominator amounted to 300,062. In contrast, the population count encompassed 374,800 children under one. Over 36 months, the total number of RTS,S/AS01 vaccine doses administered was: 292,199 for RTS,S/AS01 dose 1, 254,864 dose 2, 215,343 dose 3 and, 56,242 vaccines dose 4 (Table 1). Overall, health facilities administered a median of 395 (IQR: 217–690) doses (dose 1), 342 (183–600) dose 2, and 292 (159–504) dose 3 vaccines. For dose 4 of the RTS,S/AS01 vaccine schedule at 24 months of age, the median number of doses per facility was only 75 (38–504). The highest number of vaccines administered by a single facility was 3527 vaccines of dose 1, and the lowest with only a single vaccine administered for dose 4. Facility delivery of Penta 1 and RTS,S/AS01 dose 1 vaccine exhibit similar trends with relatively similar high and low volume facilities across the two vaccines (Additional file 1: Figure S4). Facilities managed by MoH and FBOs account for over 88% of all vaccines administered across the 3 years. Private facilities contribute between 7 and 9% across all doses with NGOs and ‘other’ facilities, each accounting for 1% across all doses (Table 1). ‘Other’ facilities have higher median values of over 500 for the first three doses, but comprise < 1% of vaccinating facilities (Table 1).

**Table 1:** RTS,S/AS01 vaccine delivery statistics over 36 months: total, median (IQR), and range (min–max)

	N	Dose 1	Dose 2	Dose 3	Dose 4
Total doses					
Overall	537	292,199	254,864	215,343	56,242
Level (% of total)					
Primary facilities	485	222,287 (76)	193,577 (76)	163,328 (76)	42,959 (76)
Secondary facilities	52	69,912 (24)	61,287 (24)	52,015 (24)	13,283 (24)
Owner (% of total)					
MoH^a^	388	226,320 (77)	197,841 (78)	165,925 (77)	45,558 (81)
Private^b^	79	25,047 (9)	21,363 (8)	18,538 (9)	4037 (7)
FBO^c^	52	33,858 (12)	29,752 (12)	25,735 (12)	5388 (10)
NGO^d^	13	3569 (1)	2943 (1)	2598 (1)	437 (1)
Other^e^	5	3405 (1)	2965 (1)	2547 (1)	822 (1)
Median (IQR)					
Overall	537	395 (217–690)	342 (183–600)	292 (159–504)	75 (38–504)
Level					
Primary facilities	485	361 (200–617)	321 (176–527)	270 (153–443)	70 (36–117)
Secondary facilities	52	1087 (627–1827)	992 (537–1668)	866 (457–1395)	194 (89–358)
Owner					
MoH	388	422 (245–717)	360 (217–607)	309 (185–503)	86 (46–138)
Private	79	198 (69–452)	173 (55–366)	170 (59–313)	39 (14–69)
FBO	52	565 (324–821)	511 (309–778)	414 (256–636)	84 (51–122)
NGO	13	275 (119–408)	232 (101–283)	172 (91–234)	37 (17–58)
Other	5	690 (214–1096)	600 (179–904)	542 (165–781)	150 (42–236)
Range (Min–Max)					
Overall	537	3520 (7–3527)	3471 (3–3474)	3185 (3–3188)	869 (1–870)
Level					
Primary facilities	485	3042 (7–3049)	2609 (3–2612)	2228 (3–2231)	599 (1–600)
Secondary facilities	52	3517 (10–3527)	3459 (15–3474)	3183 (5–3188)	854 (16–870)
Owner					
MoH	388	3518 (9–527)	3464 (10–3474)	3181 (7–3188)	869 (1–870)
Private	79	3019 (7–3026)	2279 (3–2282)	1984 (6–1990)	462 (1–463)
FBO	52	2792 (10–2802)	2462 (15–2477)	2203 (5–2208)	538 (6–544)
NGO	13	711 (10–721)	618 (5–623)	688 (3–691)	63 (3–66)
Other	5	1311 (47–1358)	1212 (35–1247)	987 (36–1023)	388 (3–391)

Facility ownership is categorized as ^a^MoH Minsitry of Health, ^b^Private (private-for-profi)t, ^c^FBO (faith based organizations), ^d^NGO (non-governmental organizations) and ^e^“Other” (parastatals, schools or prisons)

**Fig. 2:**
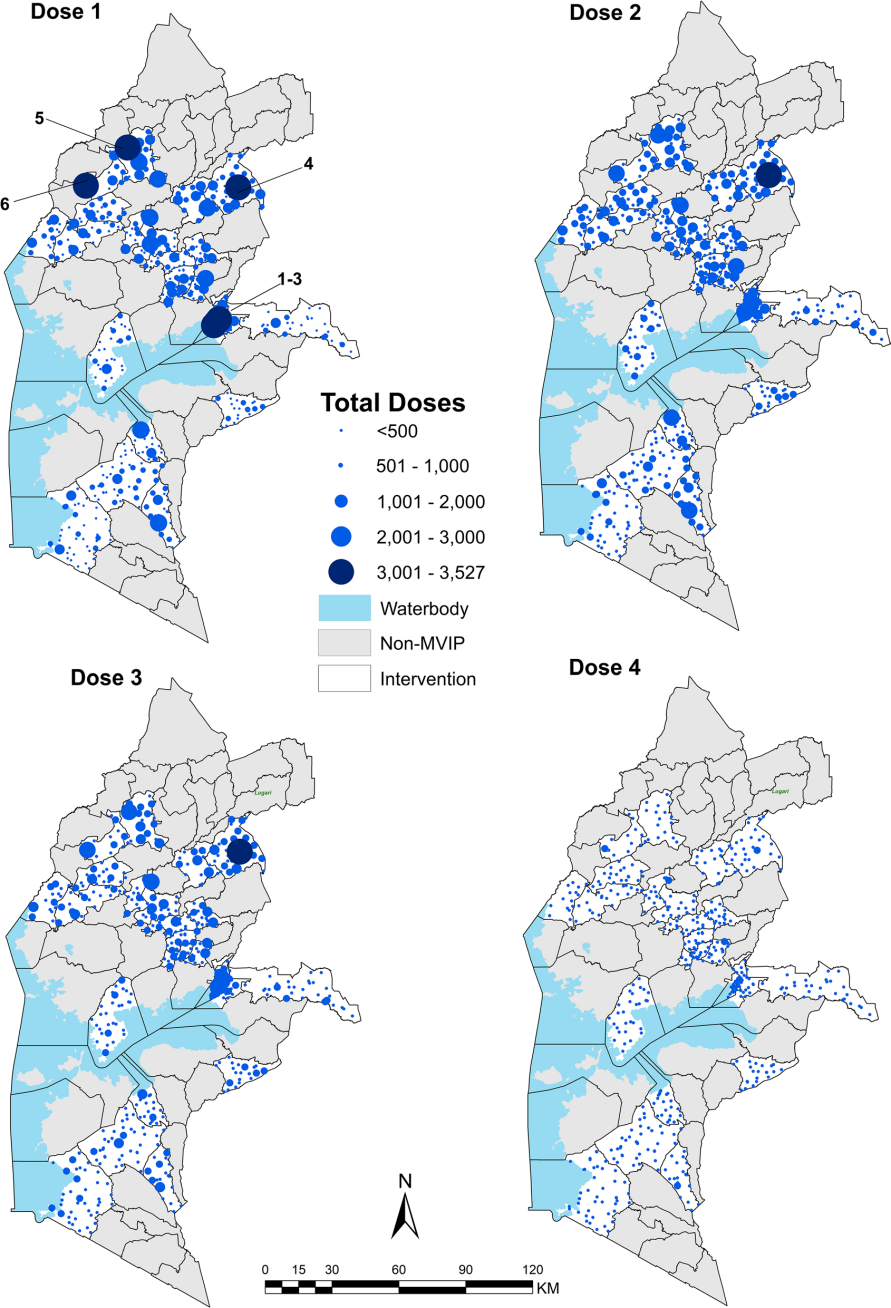
Total RTS,S/AS01 vaccine doses administered over 36 months by each health facility (N = 537). High volume (> 3000 doses) facilities: ^1^Jaramogi Oginga Odinga Teaching & Referral Hospital, ^2^Nightingale Medical Centre, ^3^Lumumba Sub County Hospital, ^4^Malava Sub County Hospital, ^5^Kimaeti Health Centre, ^6^Nambale Sub County Hospital

The uppermost quintile of vaccinating facilities consists predominantly (76%) of facilities classified as higher-level primary facilities (health and medical centres) and secondary facilities (hospitals). Markedly, secondary health facilities, which account for < 10% of vaccinating facilities (Additional file 1: Table S2) contribute nearly a quarter (24%) of total vaccines administered across all doses (Table 1). Six facilities administered over 3000 dose 1 vaccines over 3 years (Fig. 2) of which five are secondary facilities managed by MoH. Twelve secondary facilities (23%) administered over 2000 dose 1 vaccines while 99% (481) of primary health facilities administered less than 2000 doses of the first and second scheduled malaria vaccines over 36 months. The fourth dose of RTS,S/AS01 was far less often administered when it was expected to be provided following the third dose (Fig. 2).

**Fig. 3:**
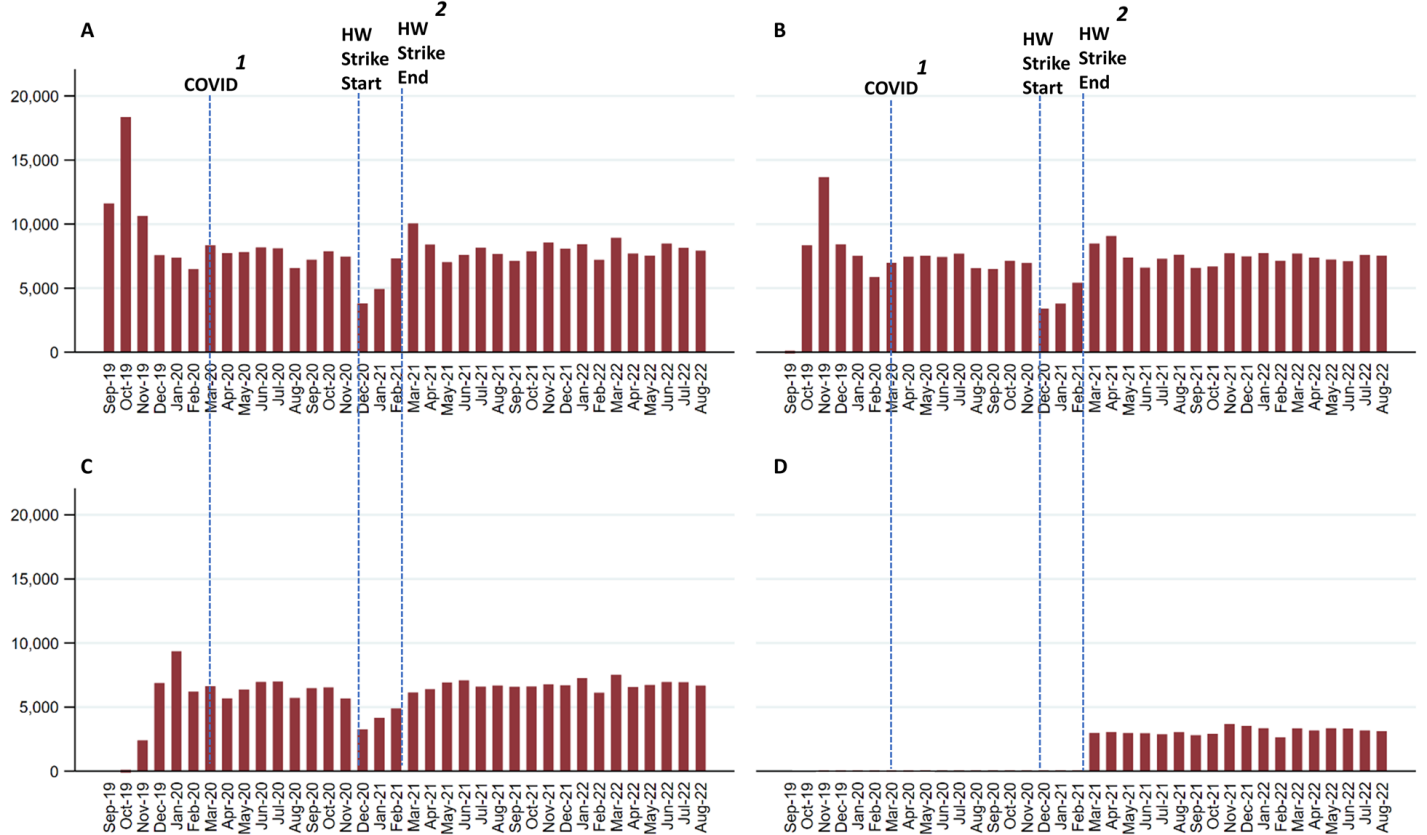
Total monthly doses of RTS, S/AS01 vaccine reported to DHIS2 (September 2019 to August 2022). ^1^First case of COVID-19 reported in Kenya,^2^National health worker strike (Dec 2020–Feb 2021)

RTS,S/AS01 delivery was at its highest in the first year with a significant peak in monthly dose 1 vaccines administered in October 2019 (18,342 vaccines), following the vaccine launch in September 2019. A similar increase in delivery was observed for dose 2 (13,663) in November 2019 and dose 3 (9349) in January 2020 as children vaccinated in October returned for subsequent doses (Fig. 3). Notably, there were decreasing totals for each respective dose denoting fewer children receiving the complete series of vaccines. The dropout rate over 36 months relative to RTSS/AS01 dose 1 was 13% for dose 2, 26% for dose 3 and 81% for dose 4. Over 36 months, delivery remained stable except for a major dip in vaccinations from December 2020 to February 2021 coinciding with national health worker strikes during the COVID pandemic (Fig. 3).

#### Coverage estimation

Using the service-based denominator (Penta 1), coverage was approximately 96% for RTS,S/AS01 dose 1, 95% dose 2, and 83% dose 3 during the first year of implementation. Coverage dropped during the second year of implementation to 90% dose 1, 83% dose 2 and 73% dose 3. RTS,S/AS01 dose 4 age-eligibility began in 2021, reaching only 37% of eligible Penta 1 recipients. During the third year of implementation, there was a 6% increase in coverage for dose 3 and a 3% increase for dose 4. Across the 36 months, service use estimation of coverage was 96% for RTS,S/AS01 dose 1, 87% for dose 2, 78% for dose 3 and 39% for the fourth dose (Table 2).

**Table 2:** Annual and 36-month comparison of service-based and population-based RTS,S/AS01 vaccine coverage estimates

Period	Dose 1			Dose 2			Dose 3			Dose 4		
	Total doses (numerator)	Service (Penta 1) Coverage	Population (under 1) Coverage	Total doses (numerator)	Service (Penta 1) Coverage	Population (under 1) Coverage	Total doses (numerator)	Service (Penta 1) Coverage	Population (under 1) Coverage	Total doses (numerator)	Service (Penta 1) Coverage	Population (under 1) Coverage
Year 1	108,769	~ 96%^a^	89%	87,399	~ 95%^a^	71%	63,177	~ 83%^a^	52%	–		
Year 2	87,486	90%	70%	79,632	83%	64%	70,760	73%	57%	17,887	37%	15%
Year 3	95,944	91%	75%	87,833	83%	69%	81,406	79%	64%	38,355	40%	32%
Overall	292,199	96%	78%	254,864	87%	68%	215,343	78%	57%	56,242	39%	24%

^a^Service coverage estimates in year 1 are approximations due to the limitation of this denominator in accounting for the age review implemented whereby all children under the age of 1 could receive the first dose of the RTS,S/AS01 vaccine

Using the population-derived denominator for coverage estimation, coverage was highest for RTS,S/AS01 dose 1 and 2 in the first implementation year, 89% and 71% coverage respectively. As expected from service user estimation, population coverage declined during the second implementation year (Table 2), with a 19% and 7% reduction in coverage of doses 1 and 2 respectively compared to the first year. Population coverage of RTS,S/AS01 dose 3 was 52% during the first year of implementation and increased by 5% during the second year and 12% in the third year compared to year 1 coverage. The population estimation of coverage of dose 4 was only 15% of the target population in year 2 and 32% in year 3. Across the 36 months of implementation, RTS,S/AS01 dose 1 population eligible coverage was 78%, 68% for dose 2, 57% for dose 3 and 24% for dose 4 (Table 2).

**Fig. 4:**
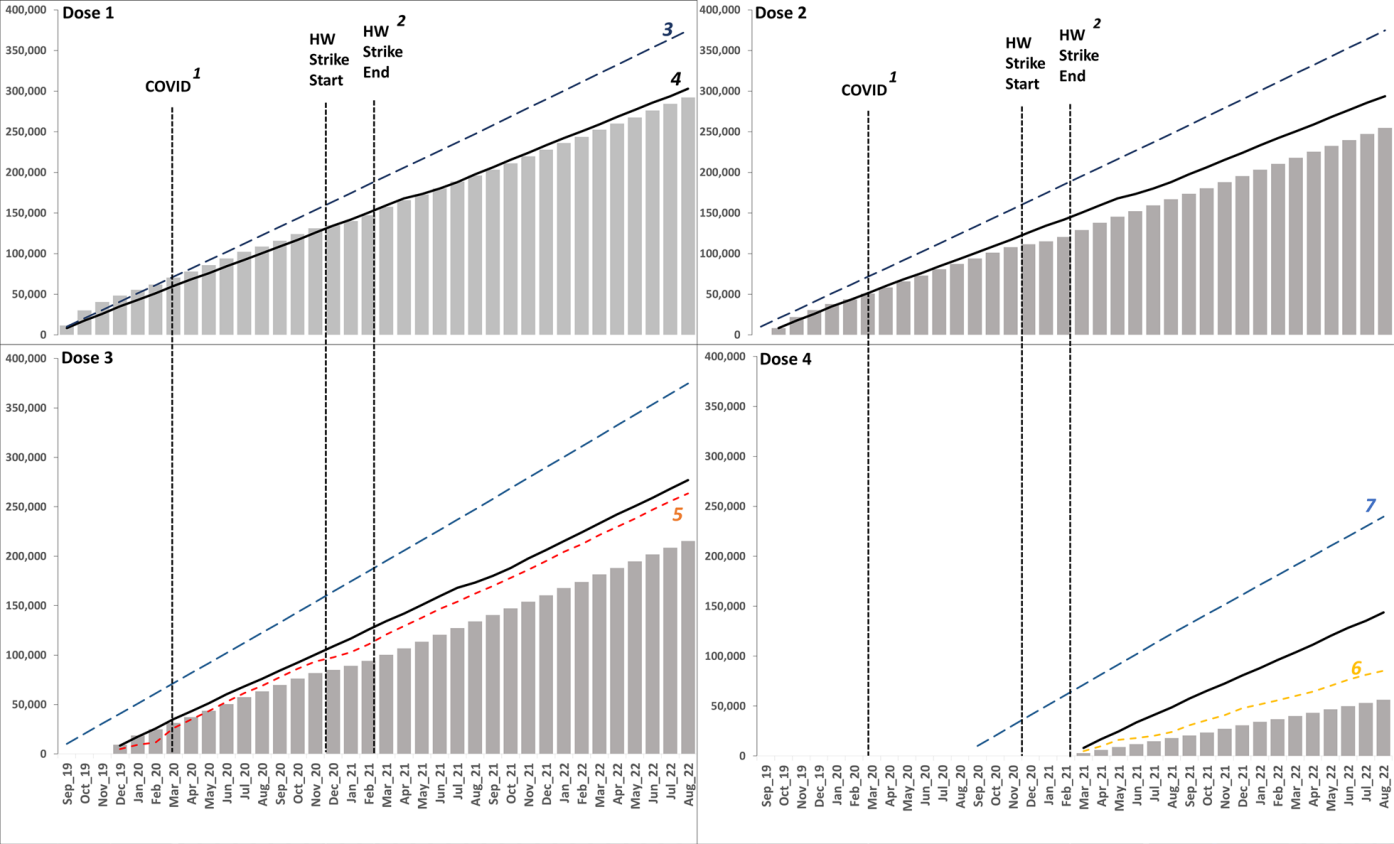
Reported cumulative doses of RTS,S/AS01 vaccines administered over 36 months relative to key infant vaccinations. ^1^First case of COVID-19 reported in Kenya, ^2^National health worker strike (Dec 2020–Feb 2021), ^3^Total Population under 1, ^4^EPI population (Penta 1), ^5^EPI population (MR1-9 months), ^6^EPI population (MR2-18 months), ^7^Total Population over 1 expected to turn 2 within the year

The results show a distinct pattern in the cumulative number of doses administered, illustrating the disparity between the number of RTS,S/AS01 doses given relative to the number of eligible recipients (Fig. 4). The first year of implementation exhibited the most successful performance for the first three doses of the malaria vaccine, as a significant portion of existing EPI users (Penta 1-eligible children) were covered. However, with increasing time from the inception of vaccine implementation and each subsequent dose, the cumulative monthly totals of all doses increasingly fell short of the EPI user and the total vaccine-eligible populations. The most significant gap was observed in the fourth dose. However, the shortfall in the third and fourth doses was lower when accounting for typical EPI drop-out rates, that is, when compared to the total MR1 and MR2 vaccinations administered. Of the total children who received MR1 vaccines (coverage: 95%) 82% (215,343/263,591) received the third dose of the RTS,S/AS01 vaccine administered at the same time. Similarly, of the total children who received MR2 (coverage:59%), 66% (56,242/85,438) received the fourth dose of the RTS,S/AS01 vaccine (Fig. 4).

### Sub-county evaluation of coverage

Sub-county level coverage rankings for RTS,S/AS01 doses 1–4 were conducted by EPI users and population denominators. The results showed significant variations in each denominator’s highest and lowest sub-county rankings. Despite similarities between the methods in identifying the same 13 sub-counties with high coverage for dose 1 and 3 and sub-counties with very low coverage for dose 4, inconsistencies were observed in the rankings for each dose between service use coverage and population-based coverage. For example, the top 3 sub-counties with the highest service use coverage for dose 2 were not among the top 3 for population-based coverage (Additional file 1: Figure S5). This is however consistent with the differences expected with the choice of denominator.

## Discussion

The present study aimed to evaluate the performance of the Kenyan implementation program for the RTS,S/AS01 vaccine over 3 years by analysing routine vaccine administration data using population and service denominators. The implementation program demonstrated high coverage (96%–78%) of RTS,S/AS01 doses 1–3 among using service users as the denominator. Conversely, using population estimates as the denominator suggested lower rates ranging from 78 to 57% of the age-eligible population receiving the first three doses. This distinction emphasizes the need to address the disparity between service users and the broader population when evaluating the overall impact of the implementation programme. Coverage was highest in the first year of implementation, and despite service interruptions through strikes, the EPI system showed some resilience and catch-up (Fig. 3). However, the programme’s success so far is tempered by the persistently low uptake of the RTS,S/AS01 dose 4 compared to those entering the EPI system (39%) (Table 2) or those remaining in the system at the time of the second Measles-Rubella vaccination (66%) and significantly lower among the infant population (24%) (Fig. 4).

The delivery of over 0.8 million doses of RTS,S/AS01 doses over 3 years demonstrates that this new vaccine can be integrated into existing EPI systems and that uptake by EPI service users was high. The highest delivery of RTS,S/AS01 doses 1 and 2 occurred during the first year of the pilot implementation programme, and delivery of these doses declined slightly during the second and third years. This could reflect the delivery becoming more routine following an initial hard push which included an expanded age criteria thereby covering children beyond the initial target population (Fig. 4). However, coverage among routine EPI service users remains imperfect. There remains poor retention of high levels of coverage following entry into the EPI schedule. The decline in RTS,S/AS01 vaccine doses across the series signals missed opportunities and incomplete vaccinations. There was a 3% difference in Penta 1 total doses administered compared to RTS,S/AS01 dose 1, and an 81% dropout in RTS,S/AS01 dose 4 compared to the first dose. This high dropout rate observed in dose 4 is similar to other late-delivery vaccines such as measles–rubella [29, 38]. Furthermore, the number of administered RTS,S/AS01 dose 3 was equivalent to 82% of the total number of MR1 vaccinations administered, suggesting that approximately 20% of children miss out on their third dose of malaria vaccination, highlighting missed opportunities for malaria vaccination for children who are likely to be presenting to facilities for other vaccines.

Phase 3 trial findings highlighted the importance of the fourth dose due to a declining immunity conferred following the first three doses [4]. Efforts are required to improve the fourth dose uptake at 24 months of age, where coverage was lowest, below 40% (EPI service users) and below 25% (eligible population). In Ghana, the challenges associated with dose 4 uptake have prompted a shift in the timing of dose 4 delivery to 18 months, aligning it with the administration of the MR2 vaccine [37]. However, it should be noted that a review of timing alone may not be adequate to achieve catch-up, as the MR2 vaccine also faces persistently high dropout rates. Additional strategies may include instituting follow-up procedures for children who miss doses and providing reminders, such as mobile text messages, to improve routine compliance [39]. Routine delivery may be further complemented by supplemental immunization activities (SIAs) as with measles and periodic mass LLIN distribution [40, 41]. Efforts to combine other late-stage vaccine schedules with mass, community-based catch-up campaigns should be explored.

It was also notable that variations in EPI delivery of RTS,S/AS01 vaccine doses was evident between facilities, primary versus secondary, ministry of Health managed versus NGO/FBO and private (Table 1). Secondary-level facilities managed by the MOH provided most of the vaccines in the implementation area. Twenty percent of the facilities in the area provided 50% of all malaria vaccine doses over the 3 years of implementation and consisted primarily of health centres and hospitals. This finding is consistent with previous studies on bypassing behaviours for maternal and child health services, whereby these facility types were often preferred [42]. This has implications for supply chain management towards ensuring the high dose vaccine centres remain stocked, but also signals further investigations on how lower order facilities and those managed by NGO and private sectors might engage more effectively in the delivery of vaccines in areas most distal to large hospital sites.

In the present analysis, two approaches to estimating malaria vaccine coverage were used, based on those who self-identified as EPI vaccine service users (Penta 1 and measles) and estimates of total children under one living within the vaccine implementation area. Each method uses data available routinely from the health information system rather than relying on periodic sample survey data on vaccine histories during household visits. However, there are constraints to both routine data methods and denominator selection. Routine DHIS2 data often has missing information and may not fully represent vaccination rates. In this study, imputation for missing data was not carried out due to several valid reasons. Firstly, vaccine administration data is generally more accurate and complete compared to other indicators. Secondly, complexities in defining facility catchments and variations in where people seek vaccinations can lead to inconsistent reporting. Additionally, differences in vaccine schedules may result in some vaccines not being administered in certain months, especially in lower-level facilities. Lastly, distinguishing between “zero reports” and genuinely missing data in DHIS2 is challenging, and using imputation could inflate vaccination figures at the facility level, potentially leading to inaccurate results. Using service-based denominators (Penta 1/MR) allows an assessment of health facility performance based on established EPI service use, enabling a measure of potential missed opportunities for new EPI antigens like the RTS,S/AS01 vaccine. However, this approach may not accurately represent community coverage as it inherently excludes children who do not attend EPI services i.e. “zero-dose” children, who are hard to enumerate through health information systems, and persistently remain under the radar and undocumented [43, 44].

Furthermore, this study makes the assumption that all vaccines are administered in accordance with the recommended EPI schedule, as illustrated in Additional file 1: Table S1. This assumption arises from the inherent limitations associated with ascertaining timeliness through routine aggregated data. It is also presupposed that children receive all vaccines in the exact sequence outlined within the schedule; for instance, it is assumed that individuals who received RTS,S/AS01 (numerator) also received Penta 1 (denominator), although this may not consistently reflect real-world scenarios. Population-based denominators estimate those eligible for vaccination within a service-providing area. As mentioned earlier, there are marked differences in the volumes of vaccines provided (Fig. 2; Additional file 1: Figure S4) depending on the level of the facility, suggesting some catchment areas are wider than others, and these can be hard to uniformly define [14, 45, 46]. In addition, empirical census data at fine spatial and age-structured resolutions are often unavailable or unreliable; consequently, the default is to use modelled age-structured population estimations [47, 48]. These modelled projections come with uncertainty which are not provided in the publicly accessible datasets.

The study findings were compared with a cross-sectional household survey undertaken in 2021 (year 2) assessing RTS, S/AS01 vaccine coverage for the first three doses across the implementation sub-counties shown in Fig. 1. The maternal recall and vaccine card estimation of coverage of RTS,S/AS01 dose 1 was 79% and dose 3 was 62% [7]. This sample survey provided similar results to those for doses 1 and 3, shown using the population-denominator approach in Table 2, suggesting an approximation to actual coverage using routine doses administered and populations eligible across the 23 sub-counties. This finding of relatively low community coverage of the vaccine, with approximately 1 in 5 children missing out on the vaccine entirely, highlights populations that remain under-served by malaria-specific interventions and routine health services more broadly. This is consistent with coverage of other malaria control measures, such as long-lasting insecticidal nets (LLIN) for children under 5 within the same region, which is approximately 75% [29]. Barriers to ensuring universal access to health services continue to play a role, impacting the success and contribution of new tools in the fight against malaria. The combination of under-served communities, exacerbated by dropouts across the vaccine schedule among those receiving early doses, has much more significant implications on the effectiveness and progress towards successful malaria control efforts.

In the present investigation of routine vaccine data, the use of population denominators, and to a lesser degree, the service-user denominators was complicated by selecting intervention areas within a wider control, non-implementation area. It is conceivable that residents on the borders of intervention areas, or further away from RTSS/AS01 vaccine centres would have sought the new vaccine from centres they would not usually use. Further, the post-launch review in age eligibility criteria for dose 1 in which all children under the age of 1 were deemed eligible [37] could not adequately be accounted for in this study, particularly in the service-based denominator, due to a lack of data on the proportion of children above 6 months who received the first dose. Estimates at the sub-county level must, therefore, be treated with caution [12, 30]. Some sub-counties (e.g. Kisumu Central) had > 100% malaria vaccine coverage based on population denominators and 13 sub-counties based on service user denominators, likely due to the presence of large secondary facilities that draw populations from multiple sub-counties and the expanded age criteria. Despite these limitations, both approaches are useful in assessing different aspects of vaccine delivery and can inform vaccine distribution strategies.

## Conclusion

While service use estimates suggest high uptake of RTS,S/AS01 of those who enter the EPI system, with inevitable dropout beyond 9 months, it is notable that population-based denominators suggest that there remain a significant proportion of children who are not accessing any dose of the RTS,S/AS01 vaccine (22%). These children who remain under the EPI radar may be similar to the 25% of children who are reported not to be using an LLIN. Thus, these populations may represent the largest contributors to the malaria disease and mortality burden in Western Kenya, to whom intervention reach is imperative.

### Supplementary Information

**Additional file 1: Table S1.** Kenya national Extended Programme for Immunisation (EPI) vaccine schedule. **Figure S1.** Flowchart of the selection process to determine facilities routinely offering vaccination services in RTS, S/AS01 intervention areas (23 sub-counties) from DHIS2 listing. **Figure S2.** Gantt chart illustration of numerator and denominator counts used for coverage computation. **Figure S3.** Annual population density maps of children under 1 year within RTS,S/AS01 implementation sub-counties for the years 2019-2022. **Table S2.** Characteristics of 537 vaccinating facilities within the 23 implementation sub-counties. **Figure S4.** Animation of cumulative Penta 1 and RTS,S/AS01 vaccines administered at facility level from September 2019 to August 2022 (N = 537). **Figure S5.** Chart showing sub-county coverage rankings of RTS,S/AS01 vaccine doses for each denominator.

## Data Availability

The datasets supporting the conclusions of this article are available online with authorized access provided by the Kenya Ministry of Health; DHIS2 portal (https://hiskenya.org/dhis-web-commons/security/login.action) The datasets used and/or analysed during the current study are available to others from these sources through the Ministry of Health Kenya.

## Abbreviations

BCG: Bacille Calmette–Guerin
DHIS2: District Health Information Software version 2
EPI: Expanded Programme of Immunization
FBO: Faith-Based Organizations
KMHFL: Kenya Master Health Facility List
KEPH: Kenya Essential Packages for Health
LLIN: Long-lasting insecticidal nets
MOH: Ministry of Health
NGO: Non-Governmental Organizations
OPV: Oral Polio Vaccine
MR: Measles–Rubella
NVIP: National Vaccines and Immunization Programme
SIA: Supplemental immunization activities
SSA: Sub-Saharan Africa
WHO: World Health Organization
