# Supplementary material for: Malaria vaccine coverage estimation using age-eligible populations and service user denominators in Kenya
Source: Malar J. 2023 Sep 27;22:287. doi: 10.1186/s12936-023-04721-0 (PMC10523632; doi:10.1186/s12936-023-04721-0)

**Additional File**

| Vaccine | Disease | Age Administered |
| --- | --- | --- |
| BCG (Baccille Calmette Guérin) | Tuberculosis | Birth |
| OPV (Oral Polio Vaccine) | Polio | Birth |
|  |  | 6 weeks |
|  |  | 10 weeks |
| IPV (Inactivated Polio Vaccine) |  | 14 weeks |
| Rotavirus | Rotavirus | 6 weeks |
|  |  | 10 weeks |
| PCV10 (Pneumococcal conjugate vaccine 10-valent) | Pneumonia | 6 weeks |
|  |  | 10 weeks |
|  |  | 14 weeks |
| Pentavalent (DTwP-Hib-HepB Whole cell) vaccine | Diphtheria, Pertussis, Tetanus, Hepatitis B and Hib (influenza, pneumonia) | 6 weeks |
|  |  | 10 weeks |
|  |  | 14 weeks |
| RTS,S/ASO1 vaccine* | Malaria | 6 months |
|  |  | 7 months |
|  |  | 9 months |
|  |  | 24 months |
| Vitamin A supplements | Vitamin A Deficiency | 6 months |
|  |  | 1-5 years (bi-annual interval) |
| MR (Measles-Rubella) | Measles, Rubella | 9 months |
|  |  | 18 months |

Table S1: Kenya national Extended Programme for Immunisation (EPI) vaccine schedule

*Delivered only in select subnational regions

Figure S1: Flowchart of the selection process to determine facilities routinely offering vaccination services in RTS, S/AS01 intervention areas (23 sub-counties) from DHIS2 listing.


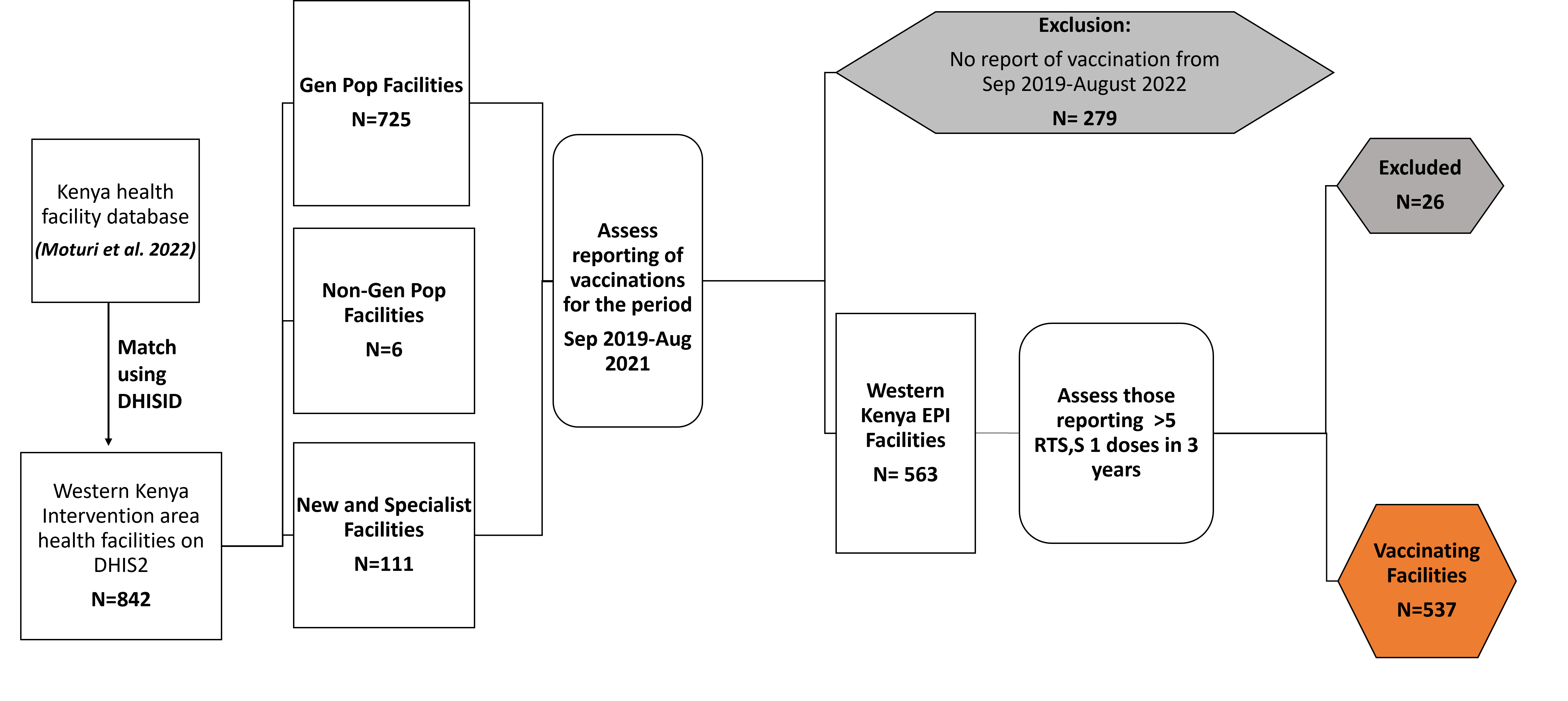


***1***

*Non-Gen Pop includes facilities such as school and army clinics that only serve select populations*

Figure S2: Gantt chart illustration of numerator and denominator counts used for coverage computation.


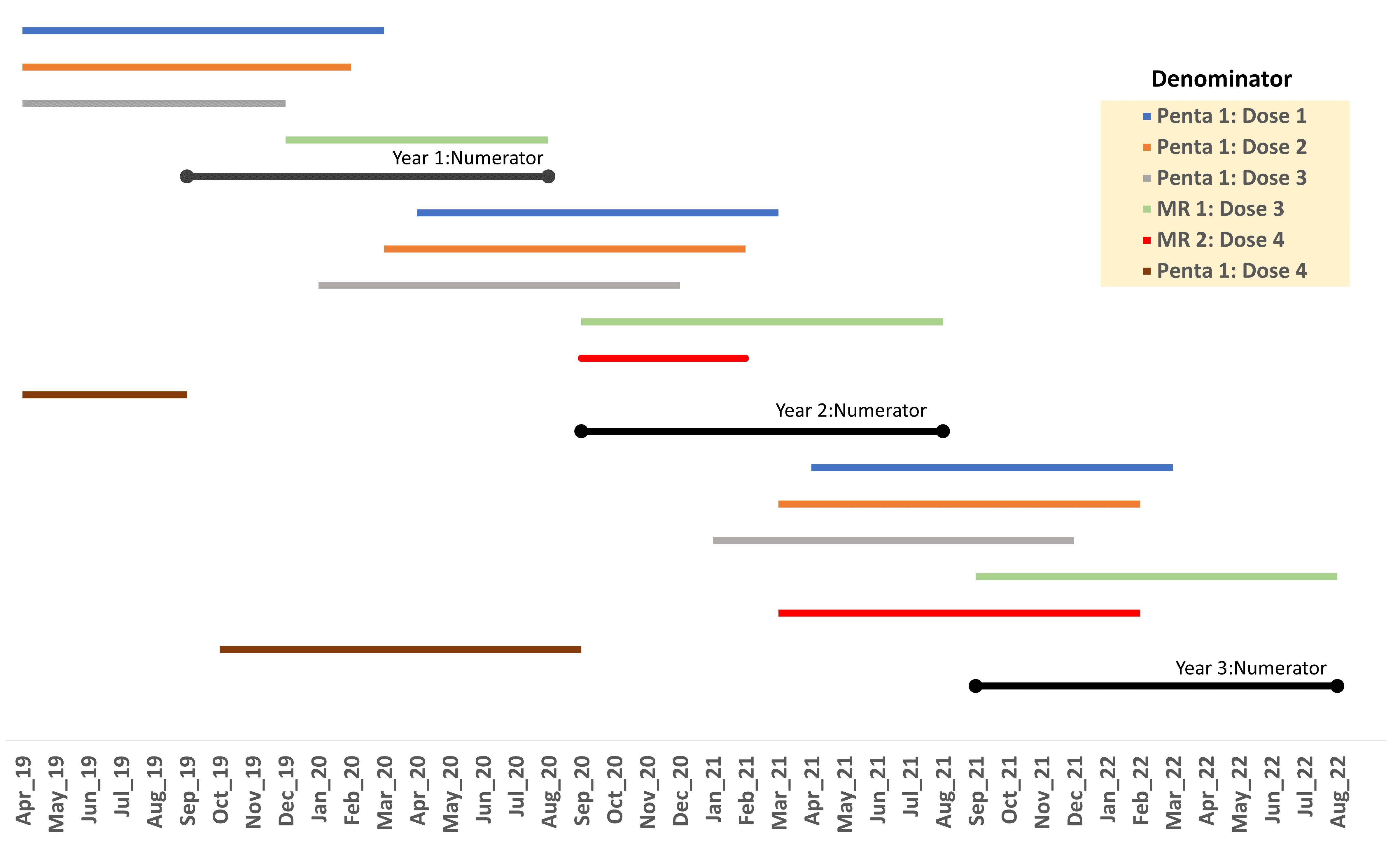


Figure S3: Annual population density maps of children under 1 year within RTS,S/AS01 implementation sub-counties for the years 2019-2022


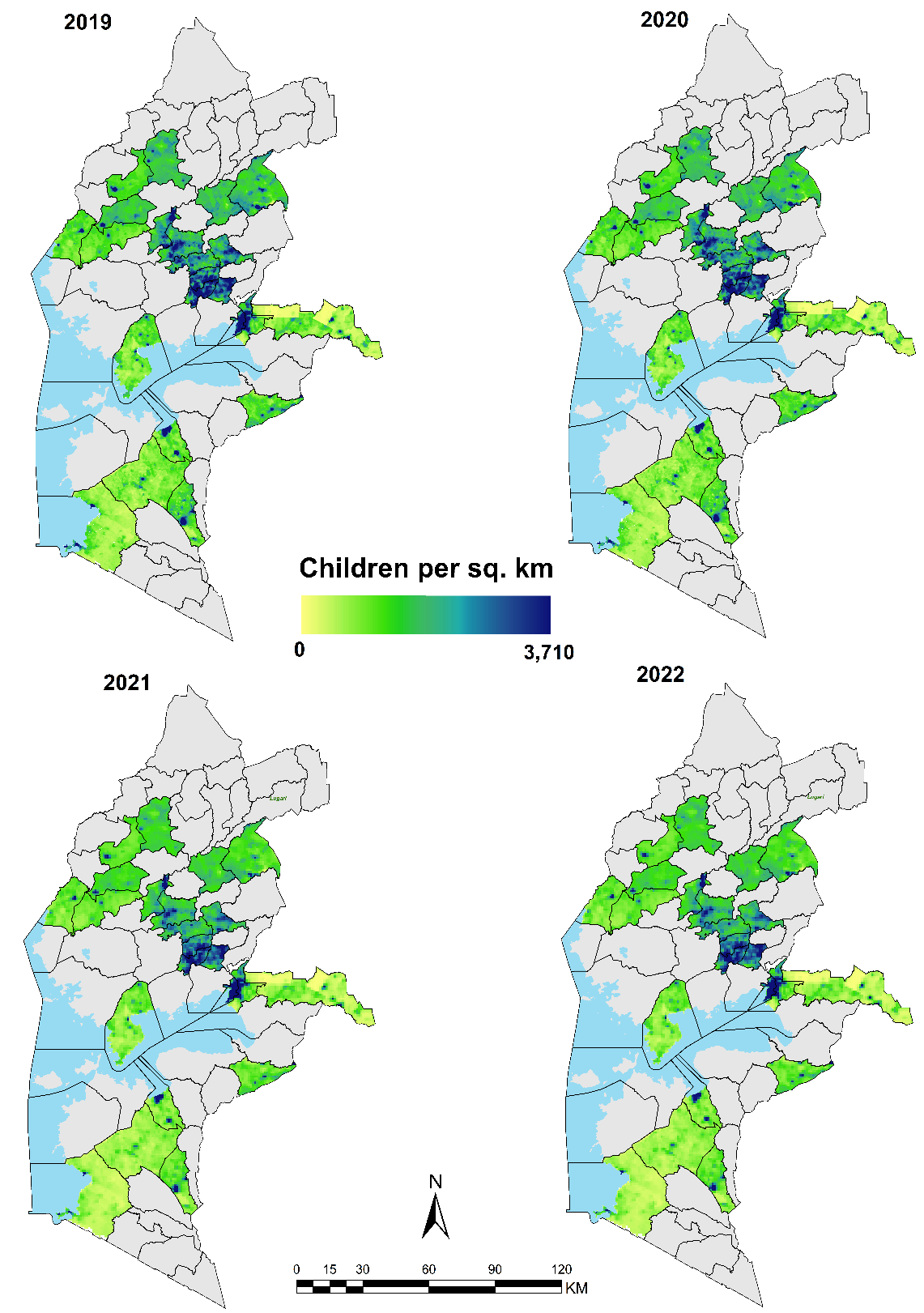


Table S2: Characteristics of 537 vaccinating facilities within the 23 implementation sub-counties

| **Category** | | **N=537** | | **%** | |
| --- | --- | --- | --- | --- | --- |
| **Owner** | |  | |  | |
| MoH | | 388 | | 72.3 | |
| Private | | 79 | | 14.7 | |
| FBO | | 52 | | 9.7 | |
| NGO | | 13 | | 2.4 | |
| Other | | 5 | | 0.9 | |
|  | |  | |  | |
| **Type** | |  | |  | |
| Clinic | | 37 | | 6.9 | |
| Dispensary | | 294 | | 54.7 | |
| Health Centre | | 122 | | 22.7 | |
| Hospital | | 53 | | 9.9 | |
| Maternity & Nursing Home | | 12 | | 2.2 | |
| Medical Centre | | 19 | | 3.5 | |
|  | |  | |  | |
| **Level of care** | |  | |  | |
| Primary | | 485 | | 90.3 | |
| Secondary | | 52 | | 9.7 | |

*Other include facilities such as government prisons, schools and parastatals that serve select populations.*

Figure S4: Animation of cumulative Penta 1 and RTS,S/AS01 vaccines administered at facility level from September 2019 to August 2022 (N=537)


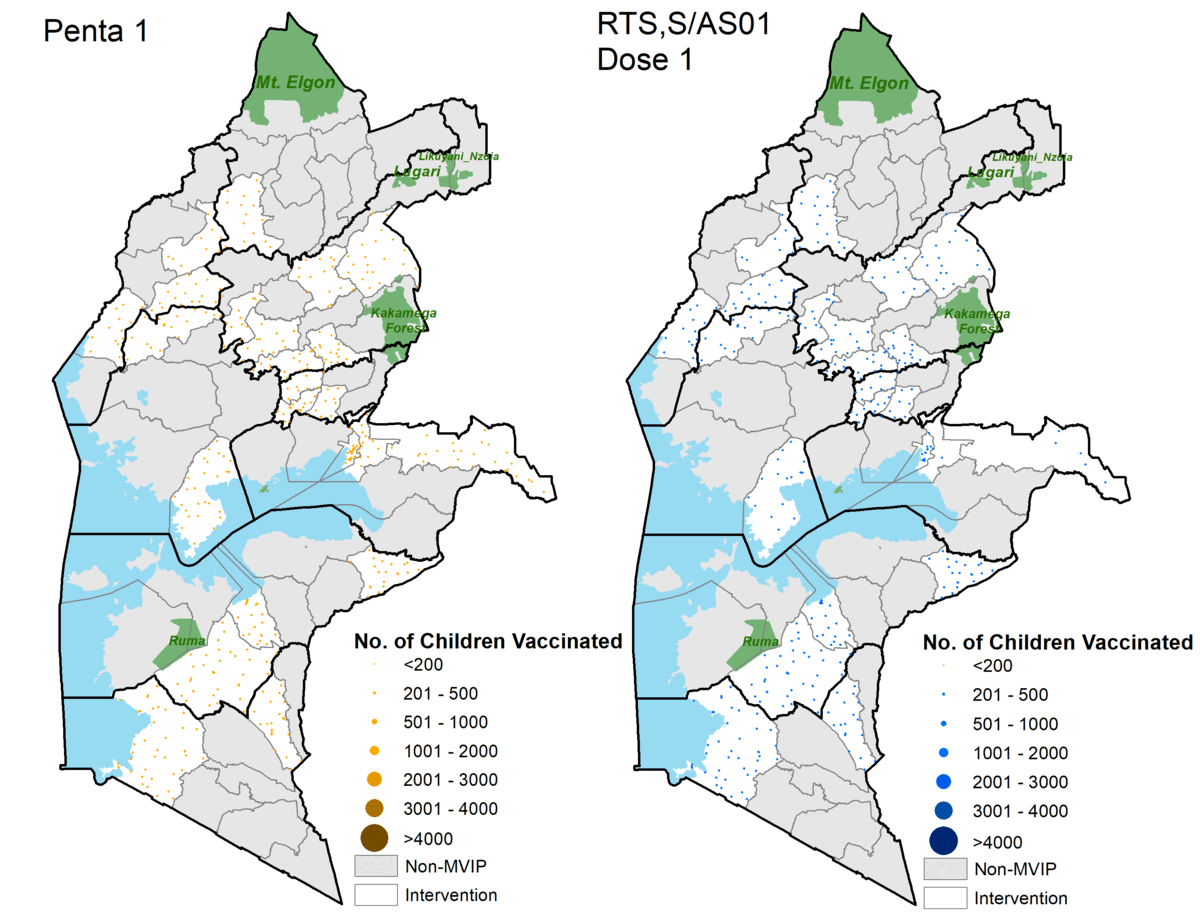


Figure S5: Chart showing sub-county coverage rankings of RTS,S/AS01 vaccine doses for each denominator


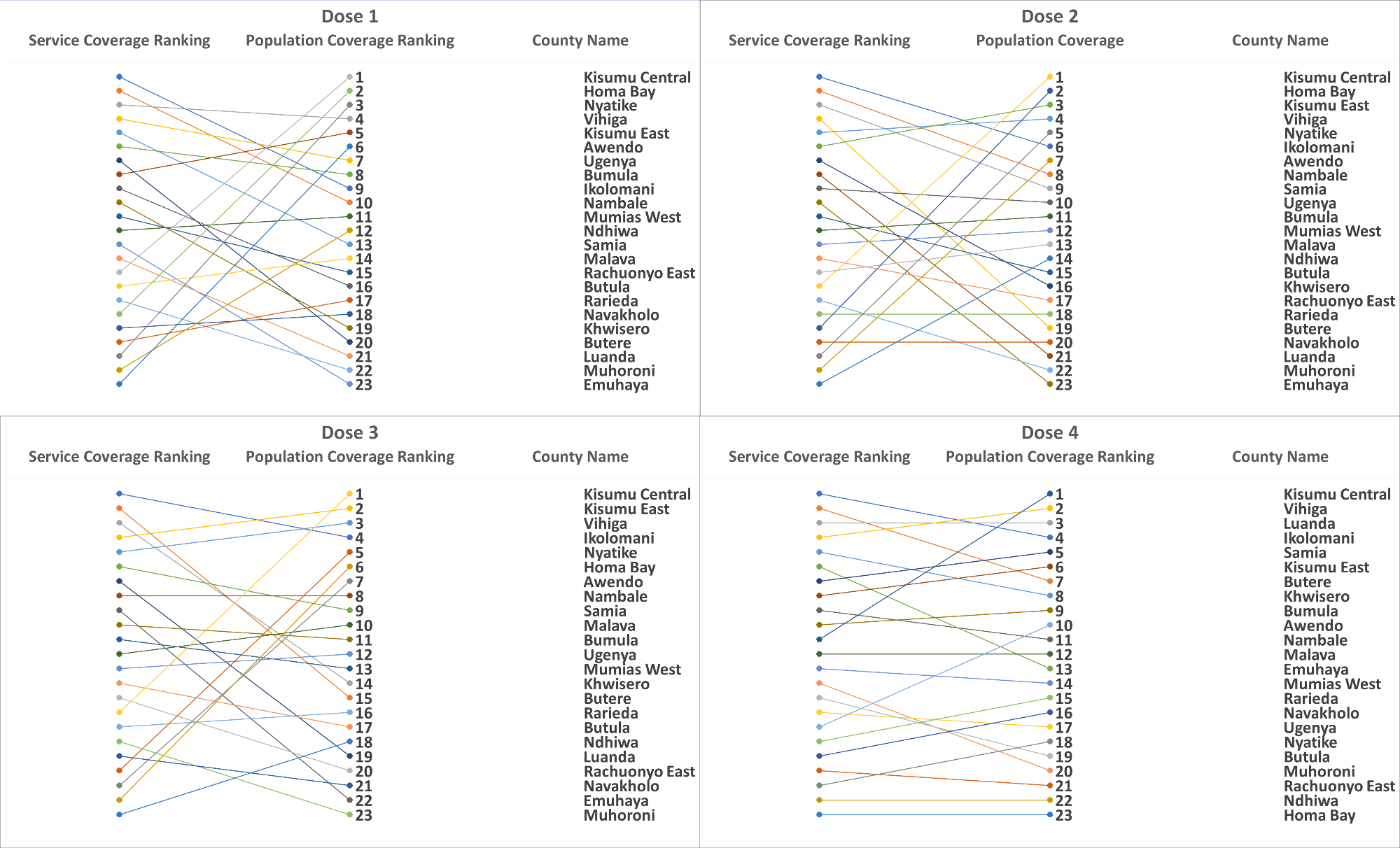

Supplement: Supplementary file 1 — Additional file 1: Table S1. Kenya national Extended Programme for Immunisation (EPI) vaccine schedule. Figure S1. Flowchart of the selection process to determine facilities routinely offering vaccination services in RTS, S/AS01 intervention areas (23 sub-counties) from DHIS2 listing. Figure S2. Gantt chart illustration of numerator and denominator counts used for coverage computation. Figure S3. Annual population density maps of children under 1 year within RTS,S/AS01 implementation sub-counties for the years 2019-2022. Table S2. Characteristics of 537 vaccinating facilities within the 23 implementation sub-counties. Figure S4. Animation of cumulative Penta 1 and RTS,S/AS01 vaccines administered at facility level from September 2019 to August 2022 (N = 537). Figure S5. Chart showing sub-county coverage rankings of RTS,S/AS01 vaccine doses for each denominator. [file 12936_2023_4721_MOESM1_ESM.docx]
